# Proactive Brain Screening Using Contrast-Enhanced Brain CT Scans in HER2+ Metastatic Breast Cancer

**DOI:** 10.1158/1078-0432.CCR-25-3557

**Published:** 2026-02-09

**Authors:** Gaia Griguolo, Michele Bottosso, Giusy Landa, Giorgio Bonomi, Federica Miglietta, Maria Cristina Guarascio, Marina La Commare, Francesca Zanghì, Terlisa Sheppard, Carlo Alberto Giorgi, Cristina Falci, Christine Hodgdon, Francesca Caumo, Maria Vittoria Dieci, Valentina Guarneri

**Affiliations:** 1 Department of Surgery, Oncology and Gastroenterology, University of Padova, Padova, Italy.; 2 Division of Oncology 2, Istituto Oncologico Veneto IRCCS, Padova, Italy.; 3 Patient Advocate, Orlando, Florida.; 4 GRASP, Baltimore, Maryland.; 5 Radiology Unit, Istituto Oncologico Veneto IRCCS, Padova, Italy.

## Abstract

**Purpose:**

According to recent EANO-ESMO guidelines, proactive brain imaging can be considered in asymptomatic patients with HER2+ metastatic breast cancer (mBC) because of high risk of developing brain metastases. However, optimal imaging modality and timing remain unclear. We retrospectively assessed the impact of contrast-enhanced CT screening on symptomatic brain metastases in patients with HER2+ mBC.

**Experimental Design:**

Consecutive patients newly diagnosed with HER2+ mBC treated with trastuzumab–pertuzumab plus taxane (2014–2024) were retrospectively identified. Brain screening was defined as at least one contrast-enhanced brain CT scan per year without neurologic symptoms during the first 2 years after diagnosis.

**Results:**

Among 148 identified patients, 73 underwent brain screening and 75 did not. The median number of annual brain CT scans during the first 2 years was 2.0 (IQR, 1.2–2.5) and 0.0 (IQR, 0–0.5) in the screening and nonscreening groups, respectively. Thirty (20.3%) patients developed brain metastases during the first 2 years. The cumulative brain metastasis incidence was significantly higher in patients undergoing screening (30.6% vs. 12.3%, Gray’s *P* = 0.004), but symptomatic brain metastases were significantly lower in patients undergoing screening (0% vs. 9.5%, Gray’s *P* = 0.012). Patients undergoing screening had better preserved performance status at brain metastasis diagnosis (*P* = 0.002) and a numerical trend toward fewer brain metastases (*P* = 0.057). Treatment patterns after brain metastasis diagnosis were similar, although whole-brain radiotherapy was used less often in the screening group (14.3% vs. 44.4%, *P* = 0.073).

**Conclusions:**

Brain screening with CT scans was associated with fewer symptomatic brain metastases and better performance status at brain metastasis diagnosis, supporting proactive imaging in HER2+ mBC. Prospective studies are warranted to define optimal timing and imaging modalities.

**Translational Relevance**

Brain metastases are a frequent complication in HER2+ metastatic breast cancer (mBC), but evidence on proactive imaging in asymptomatic patients is limited. This study provides real-world data showing that contrast-enhanced CT brain screening can reduce the occurrence of symptomatic lesions and preserve performance status. Our findings support proactive imaging by demonstrating potential benefits in reducing morbidity and possibly limiting the need for whole-brain radiotherapy, informing clinical decision-making and guideline development. These results also lay the groundwork for prospective studies to define optimal imaging modalities and timing, highlighting the significance of early detection strategies in improving outcomes for patients with HER2+ mBC.

## Introduction

Patients with HER2+ breast cancer are at high risk of developing brain metastases, with approximately 30% to 50% of patients with advanced HER2+ disease experiencing central nervous system (CNS) involvement at some point during their clinical history (1–3). The management of patients with brain metastases requires a personalized, multimodal approach, integrating locoregional treatments (i.e., neurosurgery and radiotherapy) and systemic therapy tailored based on disease characteristics (4–7).

Despite significant advancements in the management of breast cancer–related brain metastases over the last decades, brain metastases remain a major source of comorbidity and a significant cause of death for patients with breast cancer (8). The impact of brain metastases on metastatic breast cancer (mBC) treatment and prognosis, along with the detrimental effects of neurologic symptoms on quality of life, have driven growing interest in the early identification of brain metastases before the onset of neurologic symptoms, with the ultimate goal of reducing symptom burden in patients affected by breast cancer–related brain metastases and increasing the proportion of patients that can be treated with less toxic local treatment options, such as stereotactic radiotherapy.

Although current guidelines do not strongly recommend routine brain imaging in asymptomatic patients with HER2+ mBC, the most recent European guidelines (EANO-ESMO) acknowledge that proactive brain imaging can be considered in high-risk patients, such as those with HER2+ and triple-negative (TN) mBC (9). However, due to the lack of evidence, no clear indication of which radiological technique should be used or when it should be performed is currently provided. Indeed, although it is well known that magnetic resonance imaging (MRI) represents the gold standard for radiological diagnosis of symptomatic brain metastases and contrast-enhanced (CE) brain CT scan is less sensitive in this setting, there is a lack of data on their relative value in proactively screening for brain metastases in asymptomatic patients with breast cancer (9, 10). Moreover, the routine use of proactive brain MRI imaging in patients with mBC presents significant challenges in terms of resource availability and allocation and requires additional contrast media administration and increased time spent in the hospital, thus potentially negatively affecting patient quality of life. In this uncertain scenario, the pragmatic clinical approach of including evaluation of the brain in the thorax/abdomen CE CT scans to stage and reevaluate patients with metastatic HER2+/TN breast cancer has been adopted by several medical oncologists in Italy and might represent a more practical and patient-friendly approach. However, we currently lack data on the clinical impact of this strategy in reducing the frequency of symptomatic brain metastasis diagnosis.

To better assess the impact of surveillance brain imaging in neurologically asymptomatic patients, we retrospectively analyzed a homogeneous cohort of patients with metastatic HER2+ breast cancer who received trastuzumab–pertuzumab in combination with taxane and assessed the impact of brain screening using CE CT scans (performed at the discretion of the treating oncologist during the first 2 years following mBC diagnosis) on the incidence of symptomatic brain metastases and patients’ characteristics at the time of brain metastasis diagnosis.

## Materials and Methods

### Patients

Consecutive patients newly diagnosed with metastatic HER2+ breast cancer and starting first-line treatment with trastuzumab–pertuzumab plus taxane–based chemotherapy at Istituto Oncologico Veneto Padova (Italy) between February 2014 and November 2024 were included.

Inclusion criteria were histologically proven HER2+ breast cancer, histologically or radiologically proven mBC diagnosis, age ≥18 years at the time of mBC diagnosis, and having received trastuzumab–pertuzumab plus taxane–based chemotherapy as first-line systemic treatment.

Exclusion criteria were the presence of symptomatic brain metastases at the time of first mBC diagnosis and incomplete imaging data on the first 2 years after mBC diagnosis.

Demographic, clinicopathologic, and treatment data were retrospectively collected from medical charts in a dedicated database. Hormone receptor (HR) expression was determined using immunohistochemistry (IHC); positivity was defined as IHC staining in at least 1% of tumor cells. Breast cancer was considered HER2+ if scored 3+ by IHC or, in case of IHC HER2 2+ score, if HER2 amplification was observed by fluorescence *in situ* hybridization or chromogenic *in situ* hybridization (FISH/CISH).

To allow for a more homogeneous evaluation of both the radiological reevaluation approach adopted by the treating oncologist (proactive brain imaging versus no use of brain imaging in asymptomatic patients) and the clinical outcomes observed (in terms of cumulative rate of brain metastasis diagnosis, overall, and according to the presence/absence of neurologic symptoms) in patients with different follow-up time and clinical pathways, we limited the observation period of this study to the first 2 years following the diagnosis of mBC.

Therefore, data on brain imaging procedures performed during the first 2 years following the diagnosis of metastatic breast cancer was retrospectively collected from medical charts, including radiology records, and analyzed. The observation interval of this study was defined as the first 2 years after mBC diagnosis (or until brain metastasis diagnosis, death, or last follow-up, whichever occurred first). For each patient, the number of CE brain CT scans and the number of CE brain MRI performed in the absence of neurologic symptoms during that observation interval was recorded. Subsequently, the number of CE brain CT scans performed per year in the absence of neurologic symptoms was calculated for each patient by dividing the absolute number of CE brain CT scans performed during the observation time by the duration of the observation interval in years.

For the purpose of this study, patients were classified as having undergone a proactive brain imaging strategy with CE brain CT scans if at least one brain CT scan per year was performed in the absence of neurologic symptoms during the observation time (generally as part of routine radiological reassessment).

This study was reviewed and approved by the involved Institutional Review Board and Ethics Committee. If necessary, according to local regulation, written informed consent was obtained from the participants. The study was conducted in accordance with the Declaration of Helsinki.

### Statistical analysis

Statistical analysis was performed using IBM SPSS (version 29) and R (version 4.3.3). Descriptive statistics were calculated for patients’ demographics and clinical characteristics. The χ^2^ test and Mann–Whitney U test were used to study the association between variables, as appropriate.

The cumulative incidence of brain metastases during the first 2 years after mBC diagnosis was evaluated using a competing risk methodology [Fine and Gray method (11, 12)]. In analyses that considered brain metastasis diagnosis as the event of interest, death without brain metastases was treated as a competing risk. In analyses that considered symptomatic brain metastasis diagnosis as the event of interest, death without brain metastases and diagnosis of asymptomatic brain metastases were treated as competing risks. In analyses that considered asymptomatic brain metastasis diagnosis as the event of interest, death without brain metastases and diagnosis of symptomatic brain metastases were treated as competing risks. Patients who had not experienced an event of interest or any of the competing risk events after 2 years (or at last follow-up, whichever occurred first) were censored at that time.

Overall survival (OS) after brain metastasis diagnosis was defined as the time from brain metastasis diagnosis to death from any cause or last follow-up. Patients alive without event at the cutoff date of this analysis (February 25, 2025) were censored at the date of last follow-up.

OS was estimated using the Kaplan–Meier method and reported with its 95% confidence intervals (95% CI). The log-rank test was used to compare OS between groups.

All reported *P* values were two-sided, and the significance level was set at 5% (*P* < 0.05).

## Results

### Patients’ characteristics

A total of 166 patients with stage IV HER2+ breast cancer treated with trastuzumab–pertuzumab plus taxane–based chemotherapy as first-line treatment were identified. Among them, 11 patients presented with symptomatic brain metastases at the time of first mBC diagnosis and were therefore excluded from the present study. For an additional seven patients, complete information on imaging performed during the first 2 years after mBC diagnosis was unavailable.

**Figure 1.**
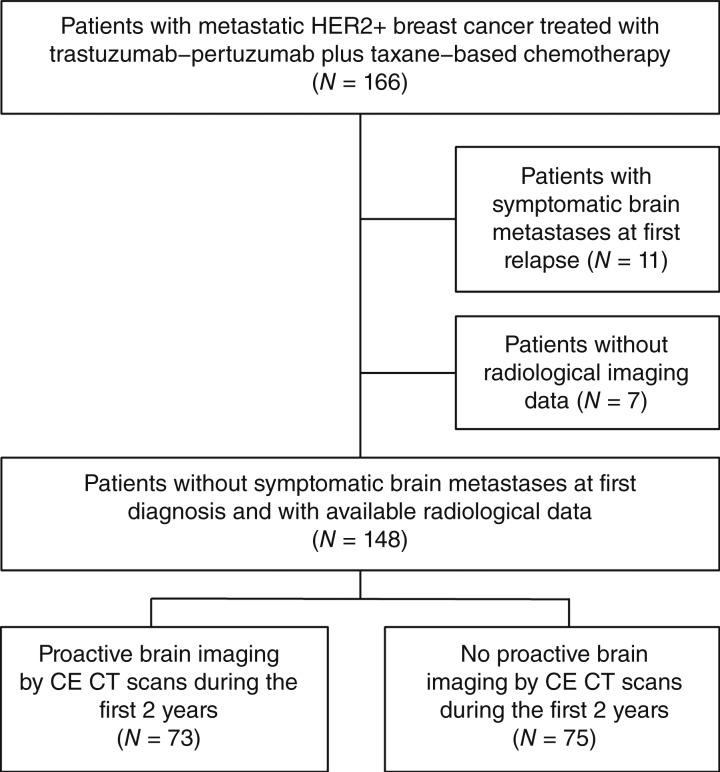
Flow diagram of the study cohort.

Among the remaining 148 patients, 73 had undergone proactive brain imaging by CE brain CT scans (at least one CE brain CT scan per year in the absence of neurologic symptoms) during the observation period of this study (first 2 years after mBC diagnosis), whereas 75 had not (Fig. 1).

Main clinicopathologic characteristics of the study cohort are reported in Table 1.

**Table 1.** Clinicopathologic characteristics at breast cancer diagnosis and at mBC diagnosis.

Characteristic	Proactive brain imaging *N* = 73 (%)	No proactive brain imaging *N* = 75 (%)	*P* value
Median follow-up after BC diagnosis (years, 95% CI)	6.0 (4.5–7.5)	9.0 (6.8–11.2)	**0.009**
Median age at BC diagnosis (range)	54 (27–82)	54 (19–79)	0.564
Age at BC diagnosis	​	​	​
≤50 years	32 (43.8)	28 (37.3)	0.421
>50 years	41 (56.2)	47 (62.7)	​
Tumor histology	​	​	0.867
No special type	62 (84.9)	65 (86.7)	
Lobular	10 (13.7)	8 (10.7)	
Other	1 (1.4)	1 (1.3)	
Not available	0 (0)	1 (1.3)	
Tumor grade	​	​	0.190
G1	0 (0)	3 (4)	
G2	16 (21.9)	13 (17.3)	
G3	54 (74)	56 (74.7)	
Not available	3 (4.1)	3 (4)	
HR status	​	​	0.519
Positive	49 (67.1)	54 (72)	
Negative	24 (32.9)	21 (28)	
Stage at BC diagnosis	​	​	​
I–III	29 (40.3)	31 (41.3)	0.896
IV	43 (59.7)	44 (58.7)	​
Year of mBC diagnosis	​	​	**<0.001**
2014 to 2017	9 (12.3)	33 (44)	
2018 to 2021	29 (39.7)	35 (46.7)	
2022 to 2024	35 (48)	7 (9.3)	
Median follow-up from mBC diagnosis (years, 95% CI)	3.0 (2.7–3.4)	5.9 (5.3–6.4)	**<0.001**
Median age at mBC diagnosis (range)	56 (28–82)	57 (19–79)	0.468
Age at mBC diagnosis	​	​	0.523
≤50 years	27 (37)	24 (32)	
>50 years	46 (63)	51 (68)	
KPS at mBC diagnosis	​	​	0.231
90–100	48 (65.8)	50 (66.7)	
70–80	18 (24.7)	21 (28)	
<70	5 (6.8)	1 (1.3)	
NA	2 (2.7)	3 (4)	
Number of metastatic organs at mBC diagnosis	​	​	0.556
1	28 (38.4)	33 (44)	
2	25 (34.2)	27 (36)	
≥3	20 (27.4)	15 (20)	
Median number of proactive brain CT scans per year (IQR)^a^	2.0 (1.2–2.5)	0.0 (0.0–0.5)	**<0.001**
Brain imaging at initial mBC diagnosis	​	​	**<0.001**
Yes	48 (65.8)	15 (20)	
No	25 (34.2)	60 (80)	
At least one brain MRI during the observation period^a^	​	​	0.063
Yes	9 (12.3)	3 (4)	
No	64 (87.7)	72 (96)	

Note: Values in bold are statistically significant.

Abbreviation: BC, breast cancer; NA, not available.

a During the observation period of the study (from mBC diagnosis to 2 years after diagnosis or until brain metastasis diagnosis, death, or last follow-up, whichever occurred first) and in the absence of neurologic symptoms. Patients with brain metastases at baseline (*N* = 6) were excluded from this analysis.

Patients undergoing and not undergoing a proactive brain imaging strategy with CE brain CT scans during the study observation period presented similar clinicopathologic characteristics at breast cancer diagnosis (Table 1). The median age at diagnosis was 54 years in both groups, most patients had grade 3 no special type breast cancer (84.9%–86.7%), around 70% presented HR+ disease (67.1%–72%), and nearly 60% of patients had *de novo* stage IV disease (59.7–58.7).

As expected, because brain screening has only recently been incorporated into guidelines, patients who underwent a proactive brain imaging strategy with CE brain CT scans during the study observation period were diagnosed significantly more recently and had a significantly shorter median follow-up from both first breast cancer diagnosis and mBC diagnosis. No significant differences were reported in terms of performance status [as evaluated by Karnofsky performance status (KPS)] and the number of metastatic sites at mBC diagnosis (*P* = 0.231 and *P* = 0.556, respectively).

As by definition, patients undergoing proactive imaging underwent a higher number of proactive (asymptomatic) brain CT scans per year during the observation period of the study (median: 2.0 vs. 0.0, *P* < 0.001). Coherently, more patients in the proactive imaging group underwent brain imaging in the absence of symptoms at the time of first mBC diagnosis compared with patients not undergoing proactive imaging (65.5% vs. 20%, *P* < 0.001). In addition, although a numerically higher number of patients undergoing proactive imaging with CE brain CT scans also performed at least one brain MRI during the observation period of this study compared with patients not undergoing proactive imaging (12.3% vs. 4%, *P* = 0.063), it should be highlighted that the use of brain MRI imaging in asymptomatic patients was extremely limited in both subgroups.

Among the 142 cases who did not present brain metastases at the time of first metastatic diagnosis, 103 (72.5%) patients were still receiving trastuzumab–pertuzumab at the time of brain metastasis diagnosis or at the 2-year cutoff (49 patients undergoing proactive brain imaging and 54 patients not undergoing proactive brain imaging), whereas 39 (27.5%) patients had progressed.

### Incidence of brain metastases

**Figure 2.**
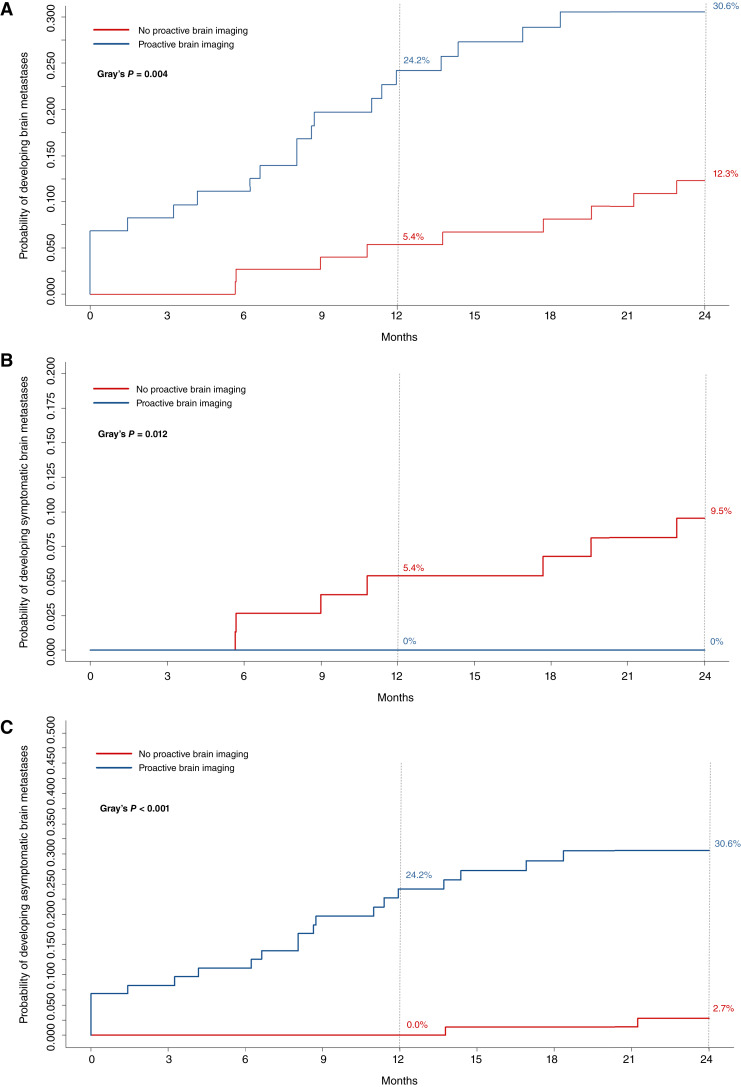
Cumulative incidence curves for diagnosis of brain metastases (**A**), diagnosis of symptomatic brain metastases (**B**), and diagnosis of asymptomatic brain metastases (**C**) according to brain screening practice.

Overall, 30 (20.3%) patients developed brain metastases within the first 2 years after mBC diagnosis, of which 21 underwent proactive brain imaging and 9 did not. Patients undergoing brain surveillance experienced an overall significantly higher cumulative incidence of brain metastases (Gray’s *P* = 0.004) at 24 months, with a 30.6% incidence compared with 12.3% among patients not undergoing brain screening (Fig. 2A).

Among these 30 patients, 7 presented neurologic symptoms at brain metastasis diagnosis, whereas 23 patients were diagnosed with brain metastases in the absence of neurologic symptoms.

No patients were diagnosed with symptomatic brain metastasis within the first 2 years after mBC diagnosis in the subgroup of patients undergoing proactive brain imaging. Coherently, patients undergoing proactive brain imaging experienced a significantly lower cumulative incidence of symptomatic brain metastases (Gray’s *P* = 0.012) at 24 months, with a cumulative incidence of 0% compared with 9.5% in the subgroup of patients who did not undergo proactive brain imaging (Fig. 2B).

On the other hand, a significantly higher cumulative incidence of asymptomatic brain metastases (Gray’s *P* < 0.001) was observed at 24 months in patients undergoing proactive brain imaging (30.6%) compared with patients who did not undergo proactive brain imaging (2.7%, Fig. 2C). Asymptomatic brain metastases were identified at the time of first metastatic diagnosis in 6 (9.5%) of the 63 patients who underwent a CE brain CT scan as part of disease restaging procedures (6/48 classified as undergoing proactive brain imaging and 0/15 classified as not undergoing proactive brain imaging). Taking into account that patients undergoing proactive brain imaging were diagnosed more recently and this might have influenced access to novel HER2-targeted agents (e.g., trastuzumab deruxtecan and tucatinib) in later lines of treatment, we conducted a subgroup analysis only in patients diagnosed with mBC between 2018 and 2021 (*N* = 64, *N* = 29 undergoing imaging, and *N* = 35 not undergoing imaging) to better isolate the effect of proactive brain imaging from the potential confounding influence of year of diagnosis. In this subgroup, proactive imaging remained associated with a numerically higher cumulative incidence of brain metastases (cumulative incidence at 1 year: 18.7% vs. 2.7%; cumulative incidence at 2 years: 30.3% vs. 13.5%, *P* = 0.131), a significantly higher incidence of asymptomatic brain metastases (cumulative incidence of asymptomatic brain metastases at 1 year: 19.0% vs. 0.0%; cumulative incidence at 2 years: 31.2% vs. 2.7%, *P* = 0.002), and a numerically lower incidence of symptomatic brain metastases (cumulative incidence of symptomatic brain metastases at 1 year: 0.0% vs. 2.7%; cumulative incidence at 2 years: 0.0% vs. 10.8%, *P* = 0.095). These findings are consistent with the results observed in the overall study cohort, although statistical power was limited by the small sample size.

### Treatments and outcome after brain metastasis diagnosis

Clinical characteristics at brain metastasis diagnosis and first treatments received for brain progression are reported in Table 2.

**Table 2.** Clinical characteristics at brain metastasis diagnosis and first treatments received (locoregional and systemic) after brain metastasis diagnosis.

Characteristic	Brain screening *N* = 21 (%)	No brain screening *N* = 9 (%)	*P* value
Neurologic symptoms	​	​	​
Yes	0 (0)	7 (77.8)	<0.001
No	100 (100)	2 (22.2)	
KPS	​	​	**0.002**
90–100	15 (71.4)	1 (11.1)	
70–80	5 (23.8)	3 (33.3)	
<70	1 (4.8)	5 (55.6)	
Median number of BM (range)	5 (1–26)	9 (1–50)	0.164
Number of BMs	​	​	0.057
1–3	10 (47.6)	1 (11.1)	
>3	11 (52.4)	8 (88.9)	
Median size of the largest BM in mm (range)	8 (4–16)	25 (5–31)	**0.040**
Extracranial status	​	​	​
CR/PR/SD	12 (80)^a^	8 (88.9)	0.571
PD	3 (20)^a^	1 (11.1)	​
First treatment after BM diagnosis	​	​	0.105
No treatment	0 (0)	1 (11.1)	
Locoregional treatment	8 (38.1)	6 (66.7)	
Locoregional treatment + change in systemic therapy^b^	2 (9.5)	1 (11.1)	
Change in systemic therapy^b^	11 (52.4)	1 (11.1)	
Neurosurgery	​	​	​
No	21 (100)	9 (100)	1
WBRT	​	​	0.073
Yes	3 (14.3)	4 (44.4)	
No	18 (85.7)	5 (55.6)	
SRS	​	​	1
Yes	7 (33.3)	3 (33.3)	
No	14 (66.7)	6 (66.7)	
Change in systemic therapy	​	​	​
Yes	7 (46.7)^a^	2 (22.2)	0.231
No	8 (53.3)^a^	7 (77.8)	​

Note: Values in bold are statistically significant.

Abbreviations: BM brain metastasis; CR, complete response; PD, progressive disease; PR, partial response; SD, stable disease; SRS, stereotactic radiotherapy.

a Excluding six patients with asymptomatic BMs diagnosed during disease staging at the time of first mBC diagnosis and who started first-line trastuzumab–pertuzumab plus taxane.

b Including start of first-line treatment with trastuzumab–pertuzumab plus taxane in patients with asymptomatic BMs diagnosed during disease staging at the time of first mBC diagnosis.

Seven patients in the non-proactive screening group presented neurologic symptoms at the time of brain metastasis diagnosis: three patients presented with vertigo and gait instability, one patient with headache, one patient with speech impairment, one patient with cranial nerve deficit, and one patient with confusion and somnolence. Of these seven patients, two (28.6%) experienced complete symptom resolution following treatment, three (42.9%) showed a partial improvement, and two (28.6%) did not show any improvement in neurologic symptoms.

In addition to a significantly higher proportion of asymptomatic cases, patients who had undergone proactive brain imaging with CE brain CT scans during the observation period of the study also presented a significantly more conserved performance status at the time of brain metastasis diagnosis, with 71.4% of them having a KPS of 90 to 100 (vs. 11.1% in patients not undergoing proactive brain imaging) and only 1 (4.8%) patient having a KPS <70 (vs. 55.6% in patients not undergoing proactive brain imaging; *P* = 0.002). Moreover, patients undergoing proactive brain imaging presented significantly smaller brain metastases at the time of first diagnosis (median size of the largest brain metastasis: 8 vs. 25 mm, *P* = 0.040), and a numerical trend toward a higher number of brain metastases was observed among patients who did not undergo brain screening (88.9% diagnosed with more than 3 brain metastases vs. 52.4%, *P* = 0.057).

No statistically significant differences in locoregional and systemic treatments (evaluated as change in systemic treatment versus continuation of the same systemic treatment) received after brain metastasis diagnosis was observed between the two subgroups. However, whole-brain radiotherapy (WBRT) was more frequently administered to patients who had not undergone proactive brain imaging, although the difference was not statistically significant (44.4% vs. 14.3%, *P* = 0.073).

With regard to systemic treatment, six patients who were diagnosed with asymptomatic brain metastases through proactive imaging at the time of first diagnosis of mBC started treatment with trastuzumab–pertuzumab and taxanes after brain metastasis diagnosis. Among the remaining 24 patients, 20 were still receiving trastuzumab–pertuzumab (alone or with chemotherapy) when brain metastases were diagnosed, whereas 4 had transitioned to second-line treatment (T-DM1 in 3 patients and trastuzumab deruxtecan in 1 patient). After brain metastasis diagnosis, 14 patients with controlled extracranial disease did not modify their systemic therapy, and brain metastases were managed exclusively with locoregional treatment, with no differences in terms of change in systemic therapy between patients undergoing or not undergoing proactive brain imaging.

**Figure 3.**
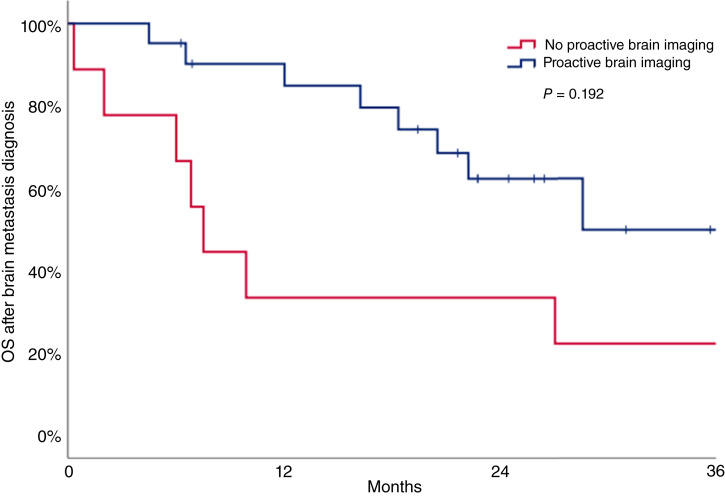
OS after brain metastasis diagnosis according to brain screening practice.

At a median follow-up after BM diagnosis of 26.4 months, 18 patients had died. The median OS after the first brain metastasis diagnosis was numerically longer among patients who underwent proactive brain imaging (28.6 months, 95% CI, 15.3–36.5) compared with patients not undergoing proactive brain imaging (7.5 months, 95% CI, 5.5–9.5, *P* = 0.192; Fig. 3).

## Discussion

The higher incidence of brain metastasis in HER2+ disease and the growing availability of effective treatment options with relevant intracranial activity have increased interest in the potential use of surveillance brain imaging in neurologically asymptomatic patients with metastatic HER2+ breast cancer. Acknowledging the significant clinical impact of breast cancer–related brain metastases, the most recent European guidelines (EANO-ESMO) have started to consider the potential role of proactive brain screening for patients with HER2+ mBC (9), whereas the NCCN guidelines currently only support the use of CE brain MRI in patients with suspicious CNS symptoms (13).

In this context, our study retrospectively assessed the impact of brain screening using CE CT scans on the incidence of brain metastases, particularly symptomatic brain metastases, among patients newly diagnosed with HER2+ stage IV breast cancer. In our study cohort, brain screening with CE CT scans was significantly associated with a significantly higher incidence of asymptomatic brain metastases and a lower incidence of symptomatic brain metastases. Notably, almost 10% of patients (6/73) undergoing baseline brain imaging were found to have asymptomatic brain metastases at first metastatic HER2+ breast cancer diagnosis, highlighting the potential value of brain screening at first stage IV diagnosis. Indeed, in this study, a significant proportion of patients was diagnosed with asymptomatic brain metastases (mainly among patients undergoing brain screening), thus suggesting that a substantial fraction of patients diagnosed with HER2+ mBC may harbor clinically silent brain metastases for a considerable portion of their metastatic disease course. This might potentially represent a window of therapeutic opportunity as timely diagnosis and intervention might potentially delay or even prevent the onset of neurologically symptomatic and potentially debilitating brain involvement.

However, the implementation of proactive brain screening in asymptomatic patients with mBC remains controversial, with inconsistent recommendations throughout different guidelines, in the absence of clear evidence of OS benefit.

Nevertheless, in mBC, quality of life and symptom control represent crucial clinical endpoints, even beyond OS benefit. Early detection of asymptomatic brain metastases might allow for earlier and less invasive treatment strategies, potentially reducing symptom burden and maintaining quality of life and neurologic functionality. Previous studies have reported that patients with breast cancer (not undergoing brain screening) were more likely to present with larger and more numerous brain metastases compared with patients with non–small cell lung cancer (NSCLC; undergoing brain screening), with a higher risk of neurologic symptoms and leptomeningeal disease (14). Although the retrospective nature of our study and the lack of quality of life data do not allow to strongly support this hypothesis, we interestingly observed significantly better KPS at the time of brain metastasis diagnosis and smaller size of brain metastases in patients who underwent brain screening. Additionally, another key prognostic factor, brain metastasis number, also showed a trend favoring patients who underwent brain screening. Consistent with these findings, patients who did not undergo proactive brain imaging had a higher likelihood of receiving WBRT at brain metastasis diagnosis (44.4% vs. 14.3%), although this difference was not statistically significant. However, this last observation should be interpreted with caution as screened patients were diagnosed more recently than those who did not, thus coinciding with a more general shift from WBRT toward less toxic locoregional treatments such as stereotactic radiosurgery. Nevertheless, as lesion size is a key factor guiding local treatment decisions for brain metastases and toxicity of stereotactic radiosurgery/radiotherapy increases with lesion size, our observations indirectly suggest that proactive brain imaging may be associated with less toxic local treatment options in patients with HER2+ breast cancer diagnosed with brain metastases. Nevertheless, these associations should be interpreted with caution given the exploratory nature of the analyses and the limited size of this subgroup.

The numerically longer median OS after brain metastasis diagnosis observed in patients who underwent proactive brain imaging (28.6 vs. 7.5 months, *P* = 0.192) should not be over-interpreted as evidence that proactive brain imaging might improve OS. This numerical difference may be easily explained by lead-time bias as earlier detection of smaller or asymptomatic lesions inherently extends the interval between brain metastasis diagnosis and death without reflecting a true survival benefit. In addition, more recently diagnosed patients also potentially had broader access to new anti–HER2-targeted agents with intracranial activity, potentially further contributing to the apparent difference in OS. Therefore, the OS findings should be interpreted with caution and should not be directly attributed to the effect of proactive imaging.

With these significant limitations, the results of our study suggest that the use of proactive brain imaging for patients with metastatic HER2+ breast cancer may be associated with a reduction in symptomatic/emergency presentations and with a more preserved performance status, thus potentially ultimately resulting in better neurologic and quality-of-life outcomes. Therefore, these results warrant confirmation in independent studies.

Indeed, currently, only a limited number of recent studies, all assessing the use of CE brain MRI, have demonstrated that proactive brain imaging can detect asymptomatic brain metastases in patients with stage IV breast cancer. However, this evidence is limited to single-arm studies with small, heterogeneous patient cohorts, often including different breast cancer subtypes and varying degrees of prior systemic therapy exposure (15, 16). Ongoing trials, including randomized studies, will help further define the role of brain surveillance in this setting (NCT04030507 and NCT03881605).

Although guidelines and existing literature primarily assess MRI as the gold standard for brain metastasis screening, its clinical applicability is limited by resource constraints, longer hospitalization times, and patient anxiety related to multiple scan procedures. From a patient perspective, CT scans may be better tolerated than MRIs because of their shorter duration, less confining design, and quieter environment, which might significantly reduce the stress associated with repeated imaging. Therefore, integrating brain CT imaging into routine CT-based disease reevaluation could offer a more practical and cost-effective alternative, with a reduced impact on quality of life, on time spent in the hospital, and on potential adverse effects of contrast medium. Nevertheless, as studies directly comparing CE brain CT scan and CE brain MRI as screening techniques for asymptomatic breast cancer–related brain metastases are lacking, we cannot exclude that MRI might offer superior sensitivity or clinical benefit in this specific setting. For instance, in a prospective study including patients diagnosed with stage III NSCLC brain MRI was able to detect asymptomatic brain metastases in 5% of patients with a negative CE brain CT scan (17). However, whether brain MRI presents higher sensitivity specifically for detecting asymptomatic breast cancer–related brain metastases and, more importantly, whether increased sensitivity would translate into clinical benefit (such as an additional reduction in symptomatic brain metastases) remain to be evaluated. In this context, identifying the optimal radiological surveillance technique and timing for screening asymptomatic brain metastases in patients with breast cancer represents an important area for future research. Moreover, as a relevant critique to the implementation of proactive brain screening, even in high-risk patients, is that this might lead to unnecessary scan-related anxiety, studies assessing whether structured imaging protocols may help reduce patient distress by offering timely interventions rather than waiting for symptom onset are warranted (18).

This study has some limitations. First, its retrospective nature did not allow for complete data collection in certain patients. Second, differences in the year of breast cancer diagnosis between the two groups, with screened patients being diagnosed more recently, may have influenced treatment strategies and outcomes. Third, the retrospective nature of this study and the small size of some of the study groups warrant for a cautious interpretation of our results. Additionally, the definition of brain surveillance as at least one brain imaging per year in asymptomatic patients is arbitrary.

Nevertheless, our study also presents some notable strengths. It includes data from a relatively large and homogeneous cohort of patients all treated with first-line trastuzumab–pertuzumab, minimizing variability in systemic therapy exposure before brain metastasis diagnosis. Moreover, applying a 2-year data cutoff helped reduce the potential impact of varying follow-up durations on brain metastasis detection and the potential impact of changes in standard second-line treatment over time (the large majority of patients were still receiving first-line pertuzumab–trastuzumab at the time of brain metastasis diagnosis). Consistently, although interpretation is limited by the small sample size, the similar findings observed in the subgroup of patients diagnosed between 2018 and 2021 (treated within a narrower time window and who likely had comparable access to subsequent treatment options) further support that our results may be driven more by proactive brain imaging than by the year of diagnosis. Finally, our study’s real-world setting supports the feasibility of this surveillance strategy in clinical practice.

In conclusion, our study suggests that at least annual brain CT surveillance may be associated with a reduction in detection of symptomatic brain metastases in patients with stage IV HER2+ breast cancer. This approach is cost-effective and is minimally invasive. Prospective trials are needed to define the optimal screening strategy, surveillance interval, and prognostic implications of proactive brain monitoring in patients with advanced breast cancer.

## Data Availability

Data that support the findings of this study are available from the corresponding author upon request, pending on formalization of a Data Transfer Agreement and necessary ethical approvals, reinforcing the compliance with the EU privacy law. Further information is available from the corresponding author upon request.
